# The morphology and histology study on rabbit degenerated medial meniscus after posterior cruciate ligament rupture

**DOI:** 10.1042/BSR20181843

**Published:** 2019-01-25

**Authors:** Zhenhan Deng, Wei Luo, Shanshan Gao, Zhan Liao, Yihe Hu, Hongbo He, Can Zhang, Kanghua Li

**Affiliations:** 1 Department of Orthopaedics, Xiangya Hospital, Central South University, Changsha, Hunan, China; 2 Department of Sports Medicine, Shenzhen Second People’s Hospital, the First Hospital Affiliated to Shenzhen University, Shenzhen, Guangdong, China; 3 Department of Cardiology, University of Colorado Anschutz Medical Campus, Aurora, Colorado, U.S.A.

**Keywords:** MMP-1, MMP-13, medial meniscus, PCL rupture, TIMP-1

## Abstract

The morphology and histology changes in the medial meniscus after posterior cruciate ligament (PCL) rupture are poorly understood. Forty-eight rabbits were divided into matched mode pairs; each rabbit had an experimental side, in which the PCL was transacted, and a control side. At the 4, 8, 16 and 24 weeks after the PCL transection, each of the 12 rabbits was killed. Histology was performed to detect the expression of the tissue inhibitors of metalloproteinases-1 (TIMP-1), matrix metalloproteinase (MMP)-1 and MMP-13 in the medial meniscus. We found that medial meniscus displayed significant degenerative characteristics in morphology. The histological evaluation of the degeneration found that the expression levels of TIMP-1, MMP-1 and MMP-13 in the medial meniscus were higher in the experiment side than those in the control side (*P*<0.05). The expression of both TIMP-1 and MMP-13 was initially elevated and then decreased. The MMP-1 expression reached its peak swiftly and then maintained a relatively high level. There were clear time-dependent degenerative changes in the histology of the medial meniscus after PCL rupture. The high expression of TIMP-1, MMP-1 and MMP-13 in the cartilage may be responsible for the degeneration, and PCL rupture may trigger meniscus degradation and ultimately osteoarthritis.

## Introduction

The posterior cruciate ligament (PCL) is widely accepted to be the strongest ligament in the knee joint; it stabilizes the knee joint by restricting posterior tibial displacement [1]. The incidence of PCL damage reported in epidemiologic studies ranges from 3% to 44% of acute knee injuries [2–4], and almost 17% of them are isolated PCL injuries [5]. Joint pain, instability and functional degradation of the knee are the most common symptoms of PCL damage. Once PCL was totally ruptured, the meniscus and other structures had to compensate to maintain the normal function of the knee joint, which might result in meniscus damage and degradation and finally osteoarthritis (OA) of the knee [6,7].

The most important biochemical change in OA is the loss of collagen type II and aggrecan, a large aggregating proteoglycan [8]. Two main enzyme families are believed to be involved in the intrinsic mechanism of degenerative changes in OA: matrix metalloproteinases (MMPs), which mediate collagen type II and a broad range of other matrix components of degeneration, and the tissue inhibitors of metalloproteinases (TIMPs), which regulate the activity of these enzymes [9]. The balance between TIMP and MMP levels is vital for the pathogenic processes of OA [10]. TIMP- and MMP-related tissue damage and degradation of the cartilage have been demonstrated in previous studies [11–13]. An examination of the expression level of TIPMs and MMPs in the meniscus in a PCL rupture model may help us to understand how meniscus degeneration is induced by PCL injury and the pathogenesis of OA [14].

Our previous study found either partial or complete rupture of the PCL can increase in the radial displacement of the medial meniscus and cause degenerative changes of the medial meniscus [15]. As part of our PCL and meniscus research series, the present study investigates the morphological and histological changes and the expression levels of TIMP-1, MMP-1 and MMP-13 in the medial meniscus after a PCL rupture using a rabbit knee joint model; specifically, it examines the correlation of these expression levels with medial meniscus degeneration and may explain the mechanism of medial meniscus degeneration after PCL rupture.

## Materials and methods

### Animal model of PCL rupture

The animal experiment was carried out in accordance with relevant guidelines and regulations, and was approved by the Medical Ethics Committee of Xiangya Hospital, Central South University (Grant number: 201212067). The present study included 48 mature male rabbits (2.6 ± 0.4 kg, 6 months), housed in separated cages at 25°C and 50–60% humidity under a 12-h light–dark cycle. The animals had free access to a normal diet and fresh tap water. Surgical transection of the PCL was performed randomly to one knee and PCL of the contralateral side was exposed but not transacted [16,17]. Specifically, the rabbits were anesthetized via the intraperitoneal administration of 3% sodium pentobarbital (0.03 mg/kg) and fixed on the operating table in a supine position. The drawer test was used to examine the stability of both sides of the knee. A patellar medial incision was used to dissect the joint capsule. The patella was then put in the lateral dislocation position, and the PCL was exposed and transected at the flexion position of the knee. The articular cavity was flushed with 3% hydrogen peroxide and then normal saline. The incision was closed without fixation of the knee joint. The same surgery was conducted on the contralateral side without the PCL transection. Postoperative anti-infection procedures were intramuscular injections of penicillin (800,000 units) once per day for 7 consecutive days. Any animal with a wound infection or suspected infection was eliminated.

### Morphology

At 4, 8, 16 and 24 weeks after PCL rupture, 12 rabbits were killed at each time point. The medial meniscus of both knees were harvested and their morphological characteristics were observed, including surface flatness, color, flexibility and intactness.

### Histology

Each medial meniscus was fixed in 4% paraformaldehyde, decalcified in diethylpyrocarbonate-treated 0.2 M ethylenediaminetetraacetic acid (EDTA), dehydrated in ethanol and xylene with a grading of concentration and embedded in paraffin. Serial sections of 3 μm were collected for hematoxylin and eosin (H&E) and immunohistochemical staining. After dewaxing, dehydration and rinsing, the specimens were dyed with hematoxylin for 5 min, given a 1 min water soaking, differentiated with 1% hydrochloric acid ethanol for 30 s, given a 15 min water soaking, then dyed with 0.5% eosin for 3 min, given a distilled water soaking and finally sealed for observation after dehydration. Light microscopy was used to evaluate the histological changes of the medial meniscus sections, which were quantified with a scoring system [18,19] (Table 1).

**Table 1:** Histological scoring system of meniscus degradation

Layer	Histological characteristics	Score
Surface	Smooth	0
	Rough	1
	Local dent	2
	Fissure or breakage	3
Cartilage cells	Big nucleus, oval or fusiform, orderly arrangement, big nucleus	0
	Big nucleus, oval or fusiform, less orderly arrangement with diffusivity	1
	Big nucleus, irregular shape, disorderly arrangement	2
	Disorderly arrangement, less cells, vacuolus cell	3
	Rare cells or vacant	4
Collagen fibers	Thick, orderly arranged and compact	0
	Thick, orderly arranged and loose	1
	Unevenly thick, orderly arranged and loose	2
	Unevenly thick, disorderly arranged and loose	3

For the immunohistochemistry, the sections were dewaxed according to the previous steps. Next, they were repaired in pancreatin at 37°C for 30 min and incubated overnight. Then they were rinsed with phosphate-buffered saline (PBS) and incubated with 1:300 rabbit polyclonal antibody TIMP-1, MMP-1 or MMP-13 at 4°C overnight. After another rinse, they were incubated with rabbit IgG at 37°C for 15 min. Finally, diaminobenzidine tetrachloride (DAB) was applied for color development, and the coverslips were counterstained with hematoxylin. A Motic Images System was used to evaluate the expression intensity of TIMP-1, MMP-1 and MMP-13 in the specimens. Areas of each specimen were observed for cell number correction using light microscopy (six non-overlapping meniscus sections and at least 10 non-overlapping fields per a side of each rabbit). The results showed a positive cell rate (PCR, PCR = positive staining cell number/total cell number × 100%).

### Statistical analysis

SPSS (version 16.0 for Windows; SPSS Inc., Chicago, IL, U.S.A.) was used for the data management and statistical analysis. The data were expressed as the mean ± standard deviation (SD). Paired *t*-tests were used to evaluate the paired data. The SNK-q test (Student–Newman–Keuls test) was used to evaluate the pairwise comparison in the cases where the mean of the data met the homogeneity of variance, whereas Dunnett’s-T3 test was used in cases where the mean of the data did not meet the homogeneity of variance. The Nemenyi rank-sum test and Wilcoxon ran-sum test were used to test the nonparametric values. Differences with *P*<0.05 were considered statistically significant.

## Results

### Morphological changes

Compared with the control sides, the medial meniscus in the PCL rupture sides presented obvious degenerative characteristics (Table 2), indicating that PCL rupture may act as a progressive degenerative factor for the medial meniscus.

**Table 2:** Morphological characteristics of medial meniscus between PCL rupture groups and control groups

	Control group		PCL rupture group		
	All time-points	4 weeks	8 weeks	16 weeks	24 weeks
Structural integrity	Integrated	Integrated	Integrated	Worn free edge	Avulsion
Surface	Smooth	Smooth	Not smooth	Rough	Rough
Color	Bright white	Gray-white	Faint yellow	Faint yellow	Yellow
Elasticity	Good	Good	Slight slack	Slack	Slack

### Histological changes

**Figure 1:**
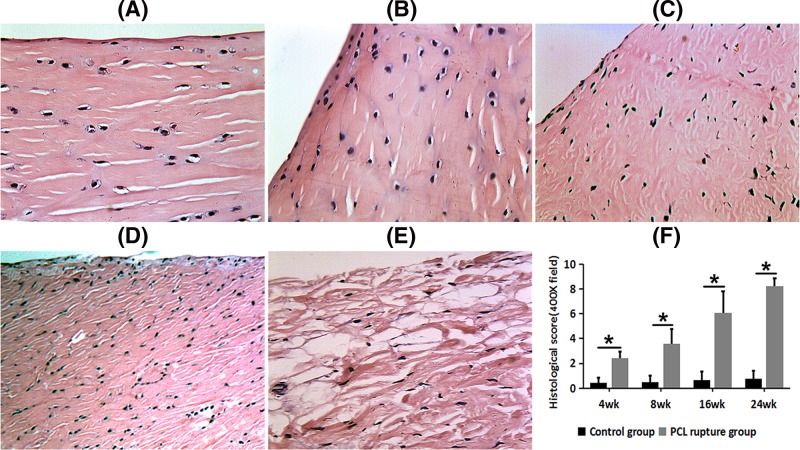
H&E staining histological characteristics in the control side and PCL rupture side of the medial meniscus at different time points (× 400 field) (**A**) The control side; (**B**) 4 weeks after PCL rupture; (**C**) 8 weeks after PCL rupture; (**D**) 16 weeks after PCL rupture; (**E**) 24 weeks after PCL rupture. (**F**) Comparison of HS between control groups and PCL rupture groups; *P<0.05.

The H&E staining of the medial meniscus in the PCL rupture sides revealed time-dependent abnormities and deterioration, whereas the collagen fibers and chondrocytes (meniscal cells) were morphologically normal on the control sides. We observed oval or fusiform shaped chondrocytes with big and round nucleus, orderly arranged thick collagen fibers in the control sides (Figure 1A). In the PCL rupture side, we could found integrate surface structure and thick, loose and orderly arranged collagen fibers at 4 weeks after PCL rupture (Figure 1B); uneven staining, rough surface, loose tissue; chondrocytes appeared disorderly arrangement and decreased in number at 8 weeks after PCL rupture (Figure 1C); rough surface with dent; collagen fibers were unevenly thick and arranged disorderly; cartilage cells significantly reduced in number and behaved disorderly arrangement at 16 weeks after PCL rupture (Figure 1D); fracture surface, loose tissue; collagen fibers were unevenly thick and arranged disorderly; meniscus cells were rarely seen at 16 weeks after PCL rupture (Figure 1E).

For the control sides, the histological scores (HS) of the medial meniscus were in the normal range, whereas the HS were much higher in the PCL rupture sides, with statistically significant differences at each time point (control group vs PCL rupture group): 0.42 ± 0.41 vs 2.41 ± 0.52 at 4 weeks after PCL rupture (*P*<0.05); 0.50 ± 0.52 vs 3.59 ± 1.16 at 8 weeks after PCL rupture (*P*<0.05); 0.67 ± 0.65 vs 6.08 ± 1.73 at 16 weeks after PCL rupture (*P*<0.05); 8.25 ± 0.62 vs 8.25 ± 0.62 at 24 weeks after PCL rupture *(P*<0.05). These results indicate that PCL rupture initiates progressive degradation of the medial meniscus (Figure 1F).

### Increased expression of TIMP-1 in the medial meniscus after PCL rupture

**Figure 2:**
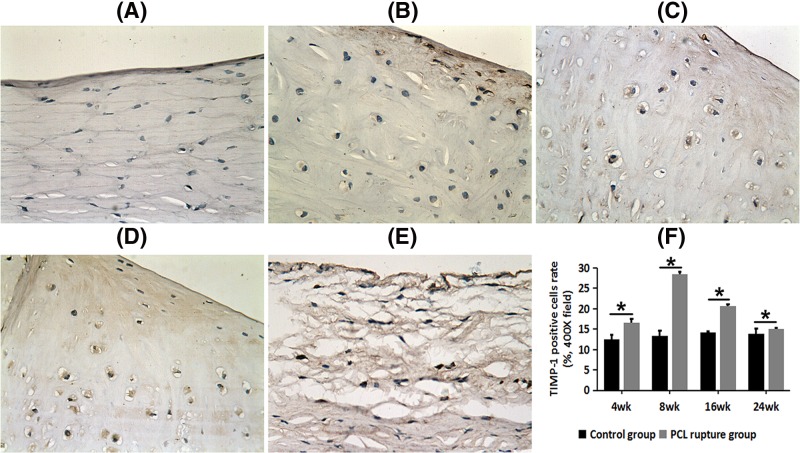
TIMP-1 expression in PCL rupture side and the control side of the medial meniscus (× 400 field) (**A**) The control side; (**B**) 4 weeks after PCL rupture; (**C**) 8 weeks after PCL rupture; (**D**) 16 weeks after PCL rupture; (**E**) 24 weeks after PCL rupture. (**F**) Comparison of PCR of TIMP-1 between control groups and PCL rupture groups; *P<0.05.

After PCL rupture, more TIMP-1 staining positive cells were observe in the medial meniscus at each time point. We could found TIMP-1 positive-staining in cytoplasm of few cells in the control side (Figure 2A). We observed limited numbers of positive-staining cells at 4 weeks after PCL rupture (Figure 2B); higher proportion of positive-staining cells 8 weeks after PCL rupture (Figure 2C); weak-staining in surface, matrix and cytoplasm at 16 weeks after PCL rupture (Figure 2D); matrix degradation, collagen fibers partly fracture, weaker staining and less positive-staining cells at 24 weeks after PCL rupture (Figure 2E).

The PCR of TIMP-1 on the PCL rupture side was significantly higher than that in the control side at every time point (*P*<0.05, Figure 2F). There was a very low level of TIMP-1 on the control side. On the rupture side, it first increased, reaching its peak at 8 weeks after PCL rupture, and then decreased in the later stages after PCL rupture.

### Increased expression of MMP-1 in the medial meniscus after PCL rupture

**Figure 3:**
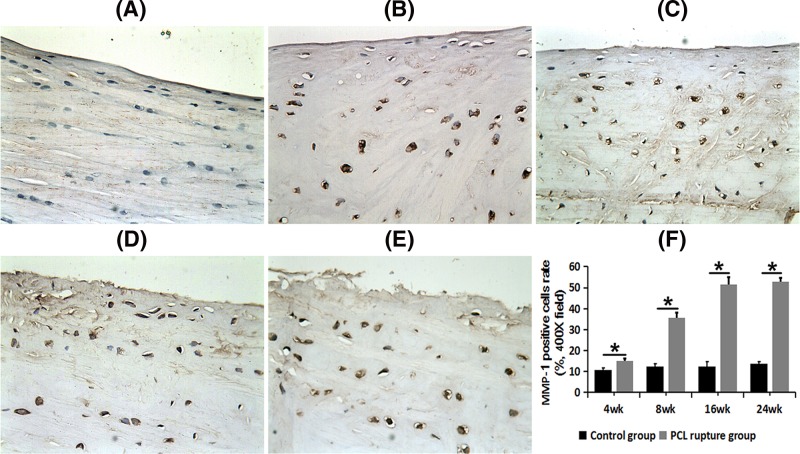
MMP-1 expression in PCL rupture side and the control side of the medial meniscus (× 400 field) (**A**) The control side; (**B**) 4 weeks after PCL rupture; (**C**) 8 weeks after PCL rupture; (**D**) 16 weeks after PCL rupture; (**E**) 24 weeks after PCL rupture. (**F**) Comparison of PCR of MMP-1 between control groups and PCL rupture groups; *P<0.05.

After PCL rupture, more MMP-1 staining positive cells were observed in the medial meniscus at each time point. We could found few MMP-1 positive staining cells in the control side (Figure 3A). We observed several oval positive cells at 4 weeks after PCL rupture (Figure 3B); higher proportion of positive-staining cells, strong positive staining in cytoplasm at 8 weeks after PCL rupture (Figure 3C); loose structure of collagen fibers, some cells deformed, strong-positive staining at both 16 and weeks after PCL rupture (Figure 3D,E).

The PCR of MMP-1 on the PCL rupture side was significantly higher than that in the control side at every time point (*P*<0.05, Figure 3F). MMP-1 was rarely detected in the control sides, whereas it showed time-dependent elevation in the cytoplasm and matrix in the PCL rupture sides of the medial meniscus. MMP-1 expression increased quickly in the early stage and then maintained a relative high level since 16 weeks after PCL rupture.

### Increased expression of MMP-13 in the medial meniscus after PCL rupture

**Figure 4:**
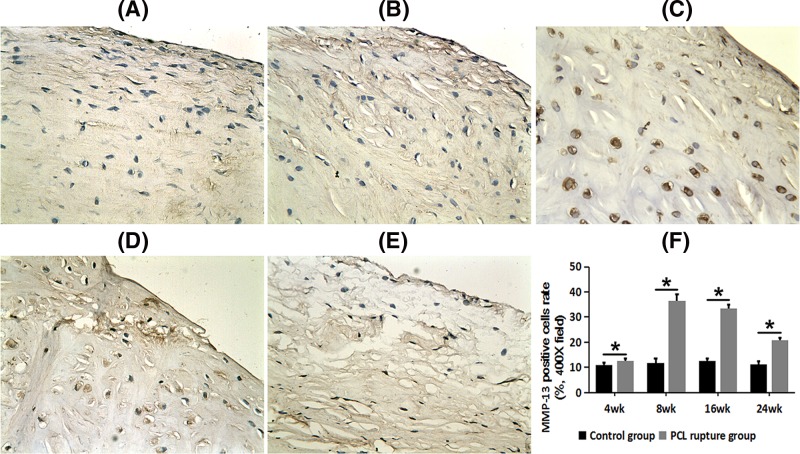
MMP-13 expression in PCL rupture side and the control side of the medial meniscus (× 400 field) (**A**) The control side; (**B**) 4 weeks after PCL rupture; (**C**) 8 weeks after PCL rupture; (**D**) 16 weeks after PCL rupture; (**E**) 24 weeks after PCL rupture. (**F**) Comparison of PCR of MMP-13 between control groups and PCL rupture groups; *P<0.05.

After PCL rupture, more MMP-13 staining positive cells were observed in the medial meniscus at each time point. We could found few MMP-13 positive-staining cells in the control side (Figure 4A). We observed a small number of positive-staining cells at 4 weeks after PCL rupture (Figure 4B); more positive-staining cells with strongly staining at 8 weeks after PCL rupture (Figure 4C); high proportion of strongly staining cells at 16 weeks after PCL rupture (Figure 4D); decreased number of positive-staining cells but still higher than the control side at 24 weeks after PCL rupture (Figure 4E).

The PCR of MMP-13 on the PCL rupture side was significantly higher than that in the control side at every time point (*P*<0.05, Figure 4F). There was a small amount of MMP-13 expression on the control sides, with an initial increase in the cytoplasm and matrix and then a low level in later stages after PCL rupture.

## Discussion

Our study design built on previous research on articular cartilage degeneration secondary to PCL rupture in rabbit knees. These studies also exposed and transected the ligament using a patellar medial incision [20]. Wang and Ao [20] found mild, moderate and severe articular cartilage damage at 6, 12 and 24 weeks after PCL rupture. Our study examined 4, 8, 16 and 24 weeks after PCL rupture as time points for medial meniscus observation; consistent with Wang’s results, our study found time-dependent meniscus tissue degeneration and a progressive degeneration after PCL rupture. No obvious signs of degradation were observed at the 4 weeks after PCL rupture, but progressive degradation was found at each subsequent time point. These morphological changes suggest that meniscus fibrous cartilage degradation concurrently after PCL rupture.

Chondrocyte is the exclusive cell type in cartilage and is approximately 5% of the total cartilage volume; the remainder is primarily extracellular matrix (ECM), which provides tension and strength to cartilage [21]. The main components of ECM are collageneous materials and aggrecans, the expression of which is mediated by MMPs [8]. Different subtypes of MMPs play different roles in various stages of cartilage degradation. As interstitial collagenases, both MMP-1 and MMP-13 can specifically decompose collagen types I, II and III, and these two types of enzymes are involved in the metabolic changes in the collagen that makes up the cartilage matrix. MMP-1, also called collagenase-1, is secreted by the cells in the lining layer of the synoviocytes and was the first MMP to be identified and purified from human fibroblasts. During the development of OA, the expression of MMP-1 is profoundly elevated in response to interleukin (IL)-1 and tumor necrosis factor-α (TNF-α) stimulation; it decomposes collagen in the ECM and causes cartilage damage, playing an important role in the disease process [22]. Collagenase-3, which is MMP-13, has the unique ability to cleave the triple helix of collagen, and participates in ECM reconstruction during the early stage of cartilage damage; it mainly targets type II collagen degradation in cartilage and is able to attend the catabolic activities induced by other members of the MMPs family. MMP-13 is widely accepted as a biochemical marker of collagenase for cartilage degeneration [23]. MMP-13 presented a high expression level associated with the expression of cartilage matrix in the early stages of OA [24]. The mechanism may be related to the dysfunction of MMP-13 receptors [25]. TIPMs are the key regulators of the inhibition of MMPs activity. TIMP-1 is the most studied TIPM; it not only inhibits the activity of activated MMPs, but also prevents and delays enzyme prototype MMPs from turning into active types [26].

Articular cartilage damage in OA is the combined effect of MMPs, cytokines and other factors. In healthy physical subjects, there is a dynamic balance between MMPs and TIMPs in the cartilage, which maintains the integrity of the cartilage structure [9]. In the early stages of OA, the human body secretes equal amounts of TIMPs and MMPs in the cartilage as part of the self-healing mechanism. Unless the abnormal load is removed or an even greater burden is added, chondrocyte degeneration becomes more apparent, resulting in the release of large amounts of catabolic cytokines and a high expression of MMPs. In the late stages of OA, a decreasing volume of catabolic cytokines and lower MMPs expression levels have been observed in articular cartilage, due to extensive damage and apoptosis of chondrocyte, but the regression continues. The imbalance between the decomposition and synthesis of the ECM reduces the cartilage matrix and destroys the structure of the cartilage; there is no positive correlation between the decrease in the matrix and cartilage degradation. The former induces apoptosis of the chondrocytes, and the dead chondrocyte cannot reproduce the cartilage matrix. As this vicious circle proceeds, the degeneration of cartilage becomes increasingly profound [27].

Previous studies of MMPs and TIMPs have focused on the cartilage, synovium and synovial fluid; little is known about their role in the meniscus. Our study examined the association between the expression of TIMP1- and MMPs and medial meniscus degeneration in a PCL rupture model. We found significantly higher MMP-1 and MMP-13 expression in chondrocytes of the transected medial meniscus than in the control group at 8 weeks after PCL rupture, and the MMP-1 expression remained high. In contrast, a dramatically low expression of MMP-13 was observed, which is consistent with other studies [28,29]. These results suggest that MMP-13 mainly functions in the early stages of OA, whereas MMP-1 plays a role during the entire progress of the disease. No consensus has been reached about TIMP-1 expression in the cartilage and synovium during OA. The majority of scholars agree that there is mild elevation or no change in expression, but Tanaka et al. [30] have argued for reduced expression. Our study found that TIMP-1 expression initially increases after surgery, but decreases during later stages, indicating that TIMP-1 has a strong role in repairing damaged cartilage at the beginning of OA, but becomes less important at later stages, perhaps due to the regulation of other mediators. The exact mechanism needs further exploration.

In conclusion, there are obvious time-dependent histological degenerative changes in the medial meniscus after PCL rupture. The high expression of MMP-1, MMP-13 and TIMP-1 in the cartilage may be responsible for the degeneration of the medial meniscus, and the PCL rupture may trigger meniscus degradation and ultimately OA.

## Availability of data and materials

All data generated or analyzed during this study are included in this published article.

## Abbreviations

ECM: extracellular matrix
EDTA: ethylenediaminetetraacetic acid
DAB: diaminobenzidine tetrachloride
H&E: hematoxylin and eosin
IL: interleukin
MMP: matrix metalloproteinase
OA: osteoarthritis
PBS: phosphate-buffered saline
PCL: posterior cruciate ligament
PCR: positive cell rate
SD: standard deviation
SNK-q test: Student–Newman–Keuls test
TIMP: tissue inhibitors of metalloproteinases
TNF-α: tumor necrosis factor-α
